# Optimization of Emulsification Conditions on Ethanol Extract of Taiwanese Green Propolis Using Polysorbate and Its Immunomodulatory Effects in Broilers

**DOI:** 10.3390/ani12040446

**Published:** 2022-02-12

**Authors:** Felix Shih-Hsiang Hsiao, Clara Ajeng Artdita, Kuo-Feng Hua, Chia-Jung Tsai, Yi-Hsuan Chien, Yue-Wen Chen, Yeong-Hsiang Cheng, Yu-Hsiang Yu

**Affiliations:** 1 Department of Animal Science and Biotechnology, Tunghai University, No. 1727, Sec. 4, Taiwan Boulevard, Taichung City 407224, Taiwan; hsh@thu.edu.tw (F.S.-H.H.); d09610702@thu.edu.tw (C.A.A.); 2 Department of Bioresources Technology and Veterinary, Vocational College, Universitas Gadjah Mada, Yacaranda 55281, Indonesia; 3 Department of Biotechnology and Animal Science, National I-Lan University, No. 1, Sec. 1, Shennong Rd., Yilan City 260, Taiwan; kuofenghua@gmail.com (K.-F.H.); chiajungtsai0101@gmail.com (C.-J.T.); yihsuanchien02@gmail.com (Y.-H.C.); chenyw@niu.edu.tw (Y.-W.C.)

**Keywords:** broilers, emulsification, immunomodulation, polysorbate, Taiwanese green propolis

## Abstract

**Simple Summary:**

Taiwanese green propolis (TGP) ethanol extract has been shown to have a wide range of biological activities, such as antimicrobial and anti-inflammatory properties. However, extraction using ethanol limits the use of TGP as an ingredient in animal feeds. In addition, the effect of TGP ethanol extract on immunomodulation in broilers is still unclear. In order to increase the utilization of TGP ethanol extract in poultry productivity, this study aimed to establish the optimal emulsification conditions for TGP ethanol extract using polysorbate and investigate its effectiveness in improving immune response in broilers.

**Abstract:**

Beeswax and resin are the main components of propolis, both of which are hydrophobic. The use of emulsifiers helps to improve the extraction of active propolis compounds and makes them more widely used. In this study, we investigated the optimal parameters for the emulsification of Taiwanese green propolis (TGP) using different polysorbates (polysorbate-20, polysorbate-60, and polysorbate-80) and evaluated the effects on the immunomodulatory response in broilers. The results showed that 4 mg/mL of TGP in combination with 2% polysorbate-60 at 60 °C for 60 min significantly decreased the undissolved particle size of ethanol extract of TGP during the emulsification. The bioactive compounds of TGP, the propolins (C, D, F, G, and H), were also detected after emulsification. Supplementation of emulsified TGP (eTGP) in the drinking water of broilers before and after vaccination significantly enhanced the antibody titer response to infectious bronchitis virus at 28 days of age. In the lipopolysaccharide-challenged model, supplementation of eTGP in the drinking water of broilers decreased pro-inflammatory gene expression and increased anti-inflammatory gene expression. These results together suggested that the polysorbate-60 could effectively emulsify the ethanol extract of TGP. Moreover, eTGP could be used as a vaccine adjuvant and an immunomodulator to improve the immune response of broilers.

## 1. Introduction

The use of antibiotic growth promoters (AGP) in animal feed has been banned by European nations since 2006, meaning researchers are still looking for AGP alternatives. Moreover, in the last decade, scientists have been developing AGP alternatives from natural products that can improve health and growth performance [1].

Propolis is a natural dark resinous substance that is processed (collected, chewed, and digested) through digestive enzymes by honeybees (*Apis mellifera*) from parts of plants (i.e., leaves, buds, fruits, and exudates). Propolis contains many beneficial compounds related to antibacterial, antioxidant, anti-inflammatory, and antitumor activities [2,3]. Likewise, propolis has been used to improve growth performance, immunity, and intestinal morphology in broilers [4,5,6,7,8].

Flavonoids and phenolic esters are the most biologically active main components of propolis [9,10,11]; the contents of these components differ depending on the method of propolis extraction [10,12]. Generally, lipophilic propolis extracted with water is used to increase the water solubility of propolis extracts. However, the contents of total flavonoids and phenolic compounds obtained by this method are very low [13]. So far, a variety of organic solvents such as ethanol have been used to extract a large number of total flavonoids and phenolic propolis compounds [10,14,15]. Nevertheless, it is worth noting that this extraction method limits the use of propolis as an ingredient in animal feeds. Emulsifiers have both hydrophilic and lipophilic substances, which can mix water and oil into a stable emulsion [16]. Polysorbate, also known as Tween, is a hydrophilic ethylene oxide polymer esterified with lipophilic fatty acids. The fatty acid connected to the polyoxyethylene sorbitan moiety determines the hydrophilic–lipophilic balance of polysorbates [17]. Polysorbates have been widely used to solubilize essential oils into water-based products, such as cosmetics, pharmaceutical solubilizers, and emulsifiers [16]. However, only a few studies have explored the effectiveness of polysorbates as emulsifiers for ethanol extracts of propolis.

Taiwanese green propolis (TGP) contains prenylated flavanone derivatives (propolins) and has been shown to have a wide range of biological activities, such as antimicrobial and anti-inflammatory properties [18,19,20]. However, the available data concerning the effects of TGP ethanol extract on immunomodulation in broilers are still scarce. Therefore, we hypothesize that the polysorbates can emulsify the TGP ethanol extract and that emulsified TGP will exhibit immunomodulatory activity in broilers. In order to improve the utilization of TGP ethanol extract in poultry production, the purpose of this study is to establish the optimal emulsification conditions for TGP ethanol extract using polysorbate and to investigate its effectiveness in improving immune response in broilers.

## 2. Materials and Methods

All of the procedures used in this experiment were approved by the Institutional Animal Care and Use Committee of National Ilan University (IACUC Approval No. 103-05).

### 2.1. Preparation of Taiwanese Green Propolis Ethanol Extract

The collection method for TGP was the same as described previously [18]. In brief, TGP was collected in a beehive from May 2015 to August 2016 in Taiwan using a propolis collector. TGP at each location from the collectors was gathered every month and kept at −20 °C until processed. To prepare the ethanol extract of TGP, the frozen TGP was ground with 80% ethanol at a concentration of 1000 µg/mL and then shaken at 250 rpm for 48 h at 25 °C. The impurities in ethanol extract were removed by filtering through Whatman no. 4 filter paper, dried in an oven, and reconstituted with 80% ethanol for the following experiments.

### 2.2. Optimization of Emulsification Conditions on Ethanol Extract of TGP

Three polysorbates (polysorbate-20, polysorbate-60, and polysorbate-80, Sigma-Aldrich, St. Louis, MO, USA) were used as emulsifiers to emulsify the ethanol extract of TGP. To screen an optimized polysorbate emulsifier for TGP, 1 mL of each polysorbate emulsifier was added to 100 mL of distilled water and stirred thoroughly. A 1% aqueous solution of different polysorbates was prepared within a triangular cone bottle, mixed well with the TGP ethanol extract to a final concentration of 2 mg/mL, heated to 60 °C, and stirred thoroughly. The mixed liquid was then emulsified in a water bath at 200 rpm and 60 °C for an hour, cooled at room temperature, and filtered with Whatman no. 3 filter paper. Since TGP will be attached to the triangular cone bottle, the filter paper and the triangular cone bottle need to be dried together in an oven at 60 °C for a further 12 h to calculate the weight of the non-emulsified TGP (the final drying weight of the filter paper and the triangular cone bottle versus the initial weight of the triangular cone bottle). The above analysis was used to examine the emulsification rate of TGP using different polysorbates. To measure the emulsification efficiency of different polysorbates (polysorbate-20, polysorbate-60, and polysorbate-80) on TGP, different percentages of polysorbates (0.25, 0.5, 0.75, 1, 1.5, and 2%) were individually mixed with TGP ethanol extract at a final concentration of 2 mg/mL. The mixture was then incubated in a water bath at 60 °C for 1 h at a speed of 200 rpm and cooled to room temperature. The emulsification efficiency of different polysorbates on TGP was measured using the absorbance at 600 nm (OD600) with a spectrophotometer. To examine the effects of the temperatures of polysorbates on TGP emulsification, 2% volumes of different polysorbates were first mixed with TGP ethanol extract (4 mg/mL) and subsequently incubated in a water bath at different emulsification temperatures (40, 50, and 60 °C) for an hour. The emulsified TGP (eTGP) was then cooled at room temperature and measured using an OD600 spectrophotometer. To further determine the time effects of polysorbates on TGP emulsification, as described above, 4 mg/mL TGP ethanol extract and 2% of each polysorbate were mixed with TGP and incubated in water baths for an hour at 40, 50, 60, and 70 °C, respectively. After 0, 20, 40, and 60 min, eTGP samples were collected, allowed to cool at room temperature, and measured using an OD600 spectrophotometer. Before the animal experiment, the individual propolin contents (C, D, F, G, and H) in the drinking water of eTGP were identified by high-performance liquid chromatography (HPLC) according to a previous study [20].

### 2.3. Animal Experiment

It has been reported that the activity levels of humoral and cell-mediated immune responses differ between the sexes of chicks during the starter phase. Therefore, the male chicks were used in the animal experiments [21]. To evaluate the vaccine adjuvant activity of eTGP and the optimal time for eTGP supplementation during vaccination, 48 one-day-old male broilers (Ross 308) were purchased from a local commercial hatchery. Chicks were housed in stainless steel cages (89 cm × 56.5 cm × 60 cm). The chicks were randomly divided into four groups and three replicates, each containing four birds (12 broilers per group), in a completely randomized design. The four groups were as follows: (1) control (Ctrl Group); (2) supplementation of 300 µg/mL eTGP in drinking water for 3 days before vaccination (eTGP was supplemented on days 1–3 and days 11–13; 300B Group); (3) supplementation of 300 µg/mL eTGP in drinking water for 3 days after vaccination (eTGP was supplemented on days 4–6 and days 14–16; 300A Group); (4) supplementation of 300 µg/mL eTGP in drinking water for 3 days before and after vaccination (eTGP was supplemented on days 1–6 and days 11–16; 300B/A Group). All broilers were vaccinated by nose-drop administration with combined Newcastle disease (ND)–infectious bronchitis (IB) live vaccines at 4 and 14 days of age. The body weight, average daily gain, average daily feed intake, and feed conversion ratio were measured at 1, 14, and 28 days of age. To evaluate the anti-inflammatory efficacy of eTGP, a total of 24 one-day-old male broiler chicks (Ross 308) were purchased from a local commercial hatchery. Chicks were randomly divided into four groups and reared individually in stainless steel cages (89 cm × 56.5 cm × 60 cm). The four groups were as follows: (1) control; (2) lipopolysaccharide (LPS, 1 mg/kg); (3) LPS plus 300 µg/mL eTGP (TGP300); (4) LPS plus 600 µg/mL eTGP (TGP600) in drinking water. The eTGP was supplied in drinking water at 18–20 days of age and LPS was administered by intraperitoneal injection at 21 days of age. All broilers were vaccinated by nose-drop administration with combined ND–IB live vaccines at 7 days of age. Diet and water were offered ad libitum. The diet was formulated according to the recommendations of the National Research Council as shown in Table 1. The temperature at the beginning was maintained at 32 °C on the first day and was reduced to 24 °C by the third week. The temperature was then maintained constantly for the remaining period of the study. The lighting schedule was 22 h light and 2 h dark throughout the experiment. All chicks were fitted with a silicone leg band that automatically loosened with leg growth.

### 2.4. Antibody Titer Analysis

Twelve broilers per group (*n* = 3) were used for ND and IB antibody titer analysis. Blood samples were collected using cardiac puncture from the same individuals for both testing. Half of the birds were used for blood collection at 14 days of age and half of the birds were used for blood collection at 28 days of age. The humoral response was assessed in terms of the determination of the antibody titer against ND and IB antigens. Briefly, blood samples were collected by cardiac puncture, left to stand at 4 °C for 12 h, and centrifuged at 1500 ×g for 10 min. Serum samples were harvested and stored in a refrigerator at −20 °C. ND and IB antibody titers were determined using the hemagglutination inhibition test and commercial enzyme-linked immunosorbent assay (ELISA) kits (Infectious Bronchitis Virus Antibody test kit, BioChek, Gouda, The Netherlands), respectively.

### 2.5. Quantitative Reverse Transcription Polymerase Chain Reaction

The broilers were euthanized using carbon dioxide inhalation. To evaluate the vaccine adjuvant activity of eTGP, total RNA samples from the spleen and bursa of Fabricius of broilers at 28 days of age were extracted using TRIzol reagent (Thermo Fisher Scientific, Waltham, MA, USA). To evaluate the anti-inflammatory efficacy of eTGP, total RNA samples from the spleen of broilers 3 h after LPS injection were extracted using TRIzol reagent (Thermo Fisher Scientific, Waltham, MA, USA). The RNA was reverse-transcribed into complementary DNA using Transcriptor reverse transcriptase kit (Roche Applied Science, Indianapolis, IN, USA). The quantitative polymerase chain reaction was performed on a MiniOpticonTM real-time PCR detection system (Bio-Rad, Hercules, CA, USA) using SYBR Green Real-Time PCR Master Mix kit (Thermo Fisher Scientific, Waltham, MA, USA). The β-actin was used as the internal control. The specific oligonucleotide primers were as follows: β-actin forward: 5′-CAT CAC CAT TGG CAA TGA GAG G-3′, and reverse: 5′-GGT ACA TTG TGG TAC CAC CAG AC-3′; tumor necrosis factor α (TNF-α) forward: 5′-CCC CTA CCC TGT CCC ACA A-3′, and reverse: 5′-TGA GTA CTG CGG AGG GTT CAT-3′; inducible nitric oxide synthase (iNOS) forward: 5′-AGG CCA AAC ATC CTG GAG GTC-3′, and reverse: 5′-TCA TAG AGA CGC TGC CAG-3′. cyclooxygenase 2 (COX-2) forward: 5′-AAC ACA ATA GAG TCT GTG ACG TCT T-3′, and reverse: 5′-TAT TGA ATT CAG CTG CGA TTC GG-3′; interleukin 4 (IL-4) forward: 5′-TGT GCC CAC GCT GTG CTT ACA-3′, and reverse: 5′-CTT GTG GCA GTG CTG GCT CTC C-3′; interleukin 10 (IL-10) forward: 5′-AGC AGA TCA AGG AGA CGT TC-3′, and reverse: 5′-ATC AGC AGG TAC TCC TCG AT-3′. Threshold cycle (Ct) values were obtained, and the relative gene expression was calculated using the formula 2^−ΔΔCt^.

### 2.6. Statistical Analysis

All experimental data were analyzed by ANOVA using the GLM procedure of SAS (SAS Institute, Cary, NC, USA). The Tukey’s HSD test was used to evaluate differences between means. Here, *p* values less than 0.05 were considered statistically significant.

## 3. Results

### 3.1. Selecting the Optimal Polysorbate Concentration for TGP Emulsification

We first determined the emulsification efficiency between different polysorbates, and 2 mg/mL TGP ethanol extract was used as test material. As shown in Table 2, the increase in emulsifier concentration improved the emulsification efficiency of the TGP ethanol extract (*p* < 0.05, Table 2). The OD600 of TGP emulsified by polysorbate-20 is significantly higher than for polysorbate-60 and polysorbate-80 at 0.25 to 1.5% concentrations (*p* < 0.05, Table 2). The increases in concentration for polysorbate-60 and polysorbate-80 dose-dependently decreased the OD600 of the ethanol extract of TGP (*p* < 0.05, Table 2). Specifically, the OD600 of polysorbate-20 at 2% concentration was not different from the polysorbate-60, indicating that the concentration of polysorbate-20 must be 2% to achieve the same TGP emulsification effect as polysorbate-60.

Further using 2% polysorbates to emulsify different concentrations of TGP (2, 4, 6, and 8 mg/mL), results showed that the OD600 of polysorbate-80 was significantly lower than forpolysorbate-20 and polysorbate-60 after emulsifying 2 mg/mL TGP (*p* < 0.05, Table 3). The effect of emulsifying 4 mg/mL TGP in 2% polysorbates-60 or polysorbates-80 was significantly higher than that in polysorbate-20 (*p* < 0.05, Table 3). No significant difference was found in emulsifying 6 and 8 mg/mL TGP between polysorbates-20, polysorbate-60, and polysorbate-80 (Table 3).

### 3.2. Effects of Different Emulsification Temperatures of Polysorbate-60 and Polysorbate-80 on TGP Emulsification

At an emulsification temperature of 40 °C, the measured OD600 of the polysorbate-60/80 group was significantly lower than the polysorbate-80 group (*p* < 0.05, Table 4). However, with increasing temperature at 50 °C, the measured OD600 values of the polysorbate-60 and polysorbate-80 groups gradually decreased (*p* < 0.05, Table 4). Interestingly, the OD600 of the polysorbate-60 group reached the lowest value at 60 °C, while the OD600 of the polysorbate-80 group increased at 60 °C and did not differ from that at 40 °C. Importantly, in the polysorbate-60/80 group, there was no significant difference in the OD600 values after TGP emulsification at different temperatures. These results suggested that 60 °C was the optimal temperature for emulsifying TGP. At this temperature, polysorbate-60 had the best ability to emulsify TGP. However, adding polysorbate-80 to polysorbate-60 may reduce the TGP emulsifying ability of polysorbate-60. Accordingly, we used polysorbate-60 at 60 °C to explore the optimal conditions for TGP emulsification in the following experiments.

### 3.3. Effects of Different Emulsification Times of Polysorbate-60 on TGP Emulsification

TGP emulsified by polysorbate-60 represented the highest OD600 at 40 °C. However, there were no differences between emulsification times (Table 5). The measured OD600 values significantly decreased with increasing emulsification time from 20 to 60 min at 50 and 60 °C (*p* < 0.05, Table 5). Specifically, the OD600 of polysorbate-60 on TGP emulsification at 50 °C was lower than that at 40 °C after 20 min of TGP emulsification. Significantly, the lowest OD600 for emulsified TGP was found at 60 °C (*p* < 0.05, Table 5); its absorbance values were gradually decreased with the time of TGP emulsification. Despite polysorbate-60-emulsified TGP having a similar OD600 profile at 60 °C and 50 °C, at each time point, the measured OD600 was significantly lower at 60 °C than those measured at 50 °C (*p* < 0.05, Table 5). At last, although TGP had the lowest OD600 at the beginning of emulsification at 70 °C, its OD600 gradually increased as the emulsification of TGP progressed (*p* < 0.05, Table 5). After 60 min of emulsification, the OD600 of emulsified TGP at 70°C was not different from that at 40 °C (Table 5). From the above results, it was suggested that treatment at 60 °C for 60 min was optimal for polysorbate-60 to emulsify the ethanol extract of TGP. These conditions were used to emulsify TGP after 80% ethanol extraction (eTGP) and for the following experiments. Furthermore, the average concentrations of propolin C, propolin D, propolin F, propolin G, and propolin H in 300 µg/mL eTGP were 0.074 mg/mL, 0.06 mg/mL, 0.029 mg/mL, 0.05 mg/mL, and 0.015 mg/mL, respectively (Table 6).

### 3.4. Evaluation of Effects of eTGP Supplementation on Immunomodulation in Broilers

**Figure 1:**
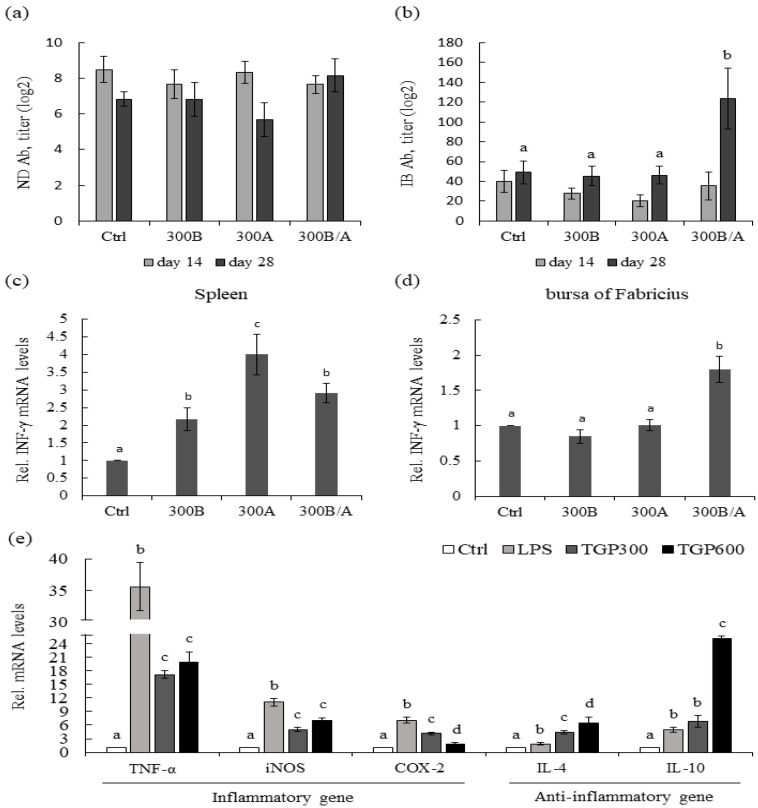
Effects of eTGP supplementation on the immunomodulatory effects in broilers. Effects of control (Ctrl) and 300 µg/mL eTGP supplemented before vaccination (300B), after vaccination (300A), and before and after vaccination (300B/A) on the serum (**a**) ND and (**b**) IB antibody titers of broilers at 14 and 28 days of age. Effects of Ctrl, 300B, 300A, and 300B/A on the IFN-γ mRNA expression in the (**c**) spleen and (**d**) bursa of Fabricius of broilers at 28 days of age. (**e**) Effects of supplementing eTGP on the various gene expression levels of TNF-α, iNOS, COX-2, IL-4, and IL-10 in the spleen of broilers challenged by LPS. Values are expressed as means ± standard deviation (*n* = 3). The means with different letter superscripts are significantly different (*p* < 0.05).

To reveal the effects of eTGP on immunity in broilers, broilers were supplemented with 300 µg/mL eTGP for 3 days before (300B group), after (300A group) and before/after (300B/A group) ND–IB vaccine administration. No dead birds were observed over the experimental period. No significant differences in growth performance were observed between groups. The effects of eTGP supplementation on vaccine adjuvant activity in broilers were then examined. The results showed that eTGP supplementation in the 300B, 300A, and 300B/A groups did not affect the ND antibody titers of 14- and 28-day-old broilers (Figure 1a). Consistently, eTGP supplementation in the 300B, 300A, and 300B/A groups did not affect the IB antibody titers of 14-day-old broilers compared to the control group (Figure 1b). Although the eTGP supplementation did not affect the IB antibody titers of 28-day-old broilers in the 300B and 300A groups, the IB antibody titer in the 300B/A group was significantly higher than that of the control group (*p* < 0.05, Figure 1b). Since IFN-γ is essential for regulating the acquired immune response; therefore, we examined whether early IFN-γ would be produced in the spleen and bursa of Fabricius of 28-day-old broilers in the 300B, 300A, and 300B/A groups after ND–IB vaccination. The supplementation of eTGP significantly increased the expression of IFN-γ mRNA in the spleen of broilers in the 300B, 300A, and 300BA groups after vaccination (*p* < 0.05, Figure 1c); the 300A group had the highest IFN-γ expression (*p* < 0.05, Figure 1c). In the bursa of Fabricius, eTGP supplementation had no effect on the expression of IFN-γ in the 300B and 300A groups. However, as compared with the control group, eTGP supplementation in the 300B/A group significantly increased the expression of IFN-γ in the bursa of Fabricius (*p* < 0.05, Figure 1d).

To reveal the immunomodulatory effects of eTGP, we examined the expression of inflammatory-associated genes caused by eTGP through the LPS challenge in broilers. Broilers were fed with 300 µg/mL (TGP300) and 600 µg/mL (TGP600) eTGP at 18–20 days of age. Subsequently, the LPS (1 mg/kg) was administered at 21 days of age in broilers. The spleen was excised 3 h after LPS injection; qPCR was used to analyze inflammatory cytokines (TNF-α, COX-2, and iNOS) and anti-inflammatory cytokines (IL-4 and IL-10). No dead birds were observed over the experimental period. No significant differences in growth performance were observed between groups. For pro-inflammatory cytokines, LPS administration significantly increased the TNF-α, COX-2, and iNOS expression levels (*p* < 0.05, Figure 1e). Three-day supplementation of TGP300 and TGP600 consistently alleviated the TNF-α, COX-2, and iNOS mRNA expression levels in the spleen (*p* < 0.05, Figure 1e). TGP300 and TGP600 showed no differences in the inhibition of TNF-α and iNOS expression levels induced by LPS. However, TGP600 can further inhibit the COX-2 expression induced by LPS compared to TGP300 (*p* < 0.05, Figure 1e). For anti-inflammatory cytokines, LPS administration significantly increased the IL-4 and IL-10 expression levels. The expression levels of LPS-induced IL-4 were significantly increased by TGP300 and TGP600 (*p* < 0.05, Figure 1e). Although TGP300 had no effect on the IL-10 expression levels, TGP600 significantly increased the IL-10 expression levels induced by LPS (*p* < 0.05, Figure 1e).

## 4. Discussion

Flavonoid has been considered as the main compound in propolis. The flavonoid content of propolis depends on the seasonal and geographical location of collection, as well often associated with various regional plants collected by honeybees. Flavonoid is a lipophilic compound. Therefore, it is easy to dissolve in ethanol. Hence, propolis is commonly extracted by ethanol and is known as the ethanolic extract of propolis [14,15]. The use of water-technology-based emulsifiers that can improve the function of active substances while being environmentally friendly still needs to be developed. Polysorbates are widely used as food- and pharmaceutical-grade emulsifiers based on the higher fatty acid esters of ethoxylated sorbitan. These are distinguished by their concentration and produce a hydrophilic–lipophilic balance (HLB) of approximately 14–18; higher HLB indicates higher hydrophilicity. Hence, this can facilitate the extraction process through the formation of micelles, which dissolve the relatively non-polar active substance of propolis into its intra-micellar cavity [22]. Our study indicated that the ideal temperature and time to emulsify TGP ethanol extracts using polysorbate-60 are 60 °C and 60 min, respectively. However, the previous study showed that using polysorbate-80 as an external phase is the best formulation to emulsify propolis, but there is no clear explanation for the temperature and time [13]. Related to the previous authors, the patent by Paradkar et al. [23] also mentioned that the formulation for emulsifying propolis is polysorbate-80 at 60 °C, but did not clearly state the duration. Based on the paper by Park and Ikegaki [24], using ethanol to extract propolis has a specific function in obtaining more compounds of kaempferide, pinocembrin, acacetin, and isorhamnetin, which strongly inhibit bacterial growth and hyaluronidase activity. Propolins, the prenylated flavonoids, are the main biologically active compounds in TGP [20]. It has been demonstrated that the average concentrations of propolin C, propolin D, propolin F, propolin G, and propolin H in TGP ethanol extracts are 35.14 mg/mL, 21.53 mg/mL, 9.7 mg/mL, 18.33 mg/mL, and 11.99 mg/mL, respectively [20]. Our study showed that the propolins (C, D, F, G, and H) were identified after the emulsification step, while the contents of propolins at concentrations of 300 µg/mL and 600 µg/mL eTGP were similar to our previous study [20]. Taken together, polysorbate-60 can be used to emulsify TGP ethanol extracts without affecting the propolin content.

It has been reported that dietary supplementation with oil-extracted propolis induces higher titers of antibodies to ND, avian influenza, and infectious bursal disease vaccines in broilers [25]. The addition of propolis in the diet of laying hens increases serum IgM and IgG levels [26]. Broilers fed propolis had a higher level of serum IgG than those fed the basal diet only and are known to increase the antigen-specific antibody response to vaccines [27]. Herein, although the antibody titers of serum ND are not affected by eTGP supplementation, this significantly increased the antibody titer of serum IB in 28-day-old broilers fed with eTGP 3 days before and after vaccination. This condition may be related to the appearance of IL-2 and IFN-γ [6]. It has been reported that IL-2 stimulates the expression of MHC type II antigens to produce IFN-γ, IL-4, IL-5, IL-6, TNF-α, TNF-β, and colony-stimulating factors [28]. Specifically, IFN-γ can induce CD4^+^, CD8^+^, T cells, and natural killer cells, additionally producing antigen presentation cells and stimulating cell proliferation. Tao et al. [29] revealed that propolis flavonoid liposome could enhance cellular and humoral immunity, in addition to inducing higher levels of IFN-γ in the serum and higher levels of splenic lymphocytes; it could also be used as an adjuvant. Consistently, the expression levels of IFN-γ in the spleen and bursa of Fabricius of 28-day-old broilers were increased in the 300B/A group during their vaccination period due to eTGP supplementation. In fact, the administration of IFN-γ and vaccine antigens in chickens is unable to enhance the immunogenicity of the ND virus vaccine [30]. This result showed that eTGP did not affect the titer of ND antibody, but significantly increased the possibility of using IB antibody titer in serum. In addition, these results indicated that supplementation of eTGP in drinking water of broilers before and after vaccination could enhance antibody titer response to the IB virus.

Compared with 14 days of age, the antibody titers of serum ND seemed to be reduced in all groups (Ctrl, 300B, and 300A) at 28 days of age, except the 300B/A group. In this current study, all broilers were vaccinated at 4 and 14 days of age and blood samples were then collected at 14 and 28 days of age. However, we did not have an unvaccinated group and did not collect blood samples before first the immunization dose for comparison. Therefore, there is no clear explanation for the reduced ND antibody titers at 28 of age; however, we can speculate that ND antibody titers may reach peak levels at 14 days of age compared with IB antibody titers.

Propolis supplementation has the ability to reduce inflammation and suppress cytokine production of immune cells [31,32]. Propolis can alleviate LPS-induced endotoxin stress by attenuating the inflammatory response and subsequent reactive oxygen species production in rats [33]. Furthermore, it has been demonstrated that propolis supplementation in laying hens can effectively reduce the inflammatory response induced by the *Escherichia coli* challenge [34]. TGP can reduce the LPS-mediated priming signal of the NLRP3 inflammasome in macrophages [18]. Here, inflammatory gene expression was reduced in the spleen of LPS challenged broilers in response to eTGP supplementation, in agreement with previous studies [33,34]. Meanwhile, anti-inflammatory gene expression was also induced in the spleen of LPS challenged broilers in response to eTGP supplementation. It has been demonstrated that IL-4 and IL-10 are immunomodulatory cytokines with a major role in inhibiting inflammation in immune-mediated pathology [35]. Taken together, supplementation of eTGP in the drinking water of broilers induces anti-inflammatory activity by modulating key pro-inflammatory and anti-inflammatory gene expression levels.

## 5. Conclusions

We demonstrated for the first time that polysorbate can be used to emulsify TGP ethanol extract and that eTGP has beneficial effects on the immunomodulatory activity and anti-inflammatory potential of broilers. The optimal emulsification conditions for emulsifying ethanol extract of TGP using polysorbates were 4 mg/mL of TGP with 2% polysorbate-60 at 60 °C for 60 min. Furthermore, eTGP could be used as a vaccine adjuvant to increase the antibody titer of IB and modulate the inflammatory-associated gene expression levels in LPS-challenged broilers. The results provide a theoretical basis for the use of TGP as a possible substitute for antibiotics in the poultry industry, while the establishment of optimal emulsification conditions for TGP ethanol extract might promote their practical application in the near future.

## Figures and Tables

**Table 1:** Nutrient composition of basal diets.

Item	Day 1 to 28
Ingredient, g kg^−1^, as-fed basis	
Corn, yellow	490.0
Soybean meal, 36.7% CP	421.8
Fish meal	50.0
CaCO_3_, 38%	20.0
CaHPO_4_	10.0
Salt	4.0
Choline, 50%	0.2
Methionine	2.0
Vitamin premix ^1^	1.0
Mineral premix ^2^	1.0
Calculated value, g kg^−1^	
Crude protein	224.5
Fat	27.2
Fiber	42.5
Calcium	13.9
Phosphorus	7.3
Lysine	13.4
Methionine + Cystine	10.0
ME, kcal/kg	3369.8

^1^ Supplied per kg diet: 1.8 mg of all-trans-retinyl acetate, 0.02 mg of cholecalciferol, 8.3 mg of alpha-tocopheryl acetate, 2.2 mg of menadione, 2 mg of pyridoxine HCl, 8 mg of cyanocobalamin, 10 mg of nicotine amid, 0.3 mg of folic acid, 20 mg of D-biotin, and 160 mg of choline chloride. ^2^ Supplied per kg diet: 32 mg of Mn (MnSO_4_·H_2_O), 16 mg of Fe (FeSO_4_·7H_2_O), 24 mg of Zn (ZnO), 2 mg of Cu (CuSO_4_·5H_2_O), 800 μg of I (KI), 200 μg of Co (CoSO_4_), and 60 μg of Se.

**Table 2:** Effects of different polysorbate emulsifiers and concentrations on the emulsification efficiency of TGP.

	Emulsifier				
Emulsifier (%)	Polysorbate-20	Polysorbate-60	Polysorbate-80	SEM	*p* Value
0.25 ^1^	1.120 ^2,x,a^	0.312 ^y,a^	0.302 ^y,a^	0.135	<0.001
0.5	0.893 ^x,b^	0.314 ^y,a^	0.255 ^y,a^	0.102	<0.001
0.75	0.868 ^x,b,c^	0.125 ^y,b^	0.093 ^y,b^	0.127	<0.001
1.00	0.708 ^x,c^	0.021 ^y,c^	0.025 ^y,c^	0.115	<0.001
1.50	0.135 ^x,d^	0.009 ^y,c^	0.011 ^y,c^	0.022	<0.01
2.00	0.005 ^x,d^	0.004 ^x,c^	0.000 ^y,c^	0.001	<0.01

Note: ^1^ 2 mg/mL TGP ethanol extract was used. ^2^ OD600 value. Values are expressed as means ± SEM (*n* = 3). ^x,y^ Means within a row with no common superscript are significantly different (*p* < 0.05). ^a–d^ Means within a column with no common superscript are significantly different (*p* < 0.05).

**Table 3:** Effects of TGP ethanol extract concentrations on the emulsification efficiency of TGP.

	Emulsifier ^1^				
TGP Ethanol Extract (mg/mL)	Polysorbate-20	Polysorbate-60	Polysorbate-80	SEM	*p* Value
2	0.005 ^2,x,a^	0.004 ^x,a^	0.000 ^y,a^	0.001	<0.01
4	1.610 ^x,b^	0.011 ^y,a^	0.022 ^y,a^	0.077	<0.001
6	1.842 ^x,c^	1.863 ^x,b^	1.804 ^x,b^	0.100	0.831
8	1.916 ^x,c^	1.852 ^x,b^	1.788 ^x,b^	0.058	0.139

Note: ^1^ 2% emulsifier was used. ^2^ OD600 value. Values are expressed as means ± SEM (*n* = 3). ^x,y^ Means within a row with no common superscript are significantly different (*p* < 0.05). ^a–c^ Means within a column with no common superscript are significantly different (*p* < 0.05).

**Table 4:** Effects of different polysorbate emulsifiers and emulsification temperatures on emulsification efficiency.

	Emulsifier ^1^				
Temperature (°C)	Polysorbate-60	Polysorbate-80	Polysorbate-60/Polysorbate-80 (1:1)	SEM	*p* Value
40	0.142 ^2,xy,a^	0.167 ^x,a^	0.125 ^y,a^	0.029	0.028
50	0.073 ^x,b^	0.094 ^xy,b^	0.143 ^y,a^	0.034	0.025
60	0.028 ^x,c^	0.145 ^y,a^	0.108 ^xy,a^	0.038	0.042

Note: ^1^ 4 mg/mL TGP ethanol extract in 2% emulsifier was tested. ^2^ OD600 value. Values are expressed as means ± SEM (*n* = 3). ^x,y^ Means within a row with no common superscript are significantly different (*p* < 0.05). ^a–c^ Means within a column with no common superscript are significantly different (*p* < 0.05).

**Table 5:** Effects of different emulsification times and temperatures on the emulsification efficiency of polysorbate-60.

	Time (Min) ^1^				
	20	40	60	SEM	*p* Value
Temperature (°C)					
40	0.297 ^2,x,a^	0.293 ^x,a^	0.294 ^x,a^	0.002	0.011
50	0.210 ^x,b^	0.184 ^y,b^	0.168 ^z,b^	0.004	<0.001
60	0.047 ^x,c^	0.026 ^y,c^	0.012 ^z,c^	0.003	<0.001
70	0.182 ^x,d^	0.217 ^x,d^	0.283 ^y,a^	0.011	<0.01

Note: ^1^ 4 mg/mL TGP ethanol extract was used. ^2^ OD600 value. Values are expressed as means ± SEM (*n* = 3). ^x–z^ Means within a row with no common superscript are significantly different (*p* < 0.05). ^a–d^ Means within a column with no common superscript are significantly different (*p* < 0.05).

**Table 6:** Determination of propolin contents in eTGP.

	Propolin C (mg/mL)	Propolin D (mg/mL)	Propolin F (mg/mL)	Propolin G (mg/mL)	Propolin H (mg/mL)
300 µg/mL eTGP	0.074 ± 0.007 ^1^	0.060 ± 0.002	0.029 ± 0.001	0.050 ± 0.004	0.020 ± 0
600 µg/mL eTGP	0.166 ± 0.003	0.151 ± 0.017	0.065 ± 0.002	0.095 ± 0.006	0.032 ± 0.001

^1^ Values are means ± SD (*n* = 3).

## Data Availability

No new data were created or analyzed in this study. Data sharing is not applicable to this article.
